# Universal‐Descriptors‐Guided Design of Single Atom Catalysts toward Oxidation of Li_2_S in Lithium–Sulfur Batteries

**DOI:** 10.1002/advs.202102809

**Published:** 2021-10-20

**Authors:** Zhihao Zeng, Wei Nong, Yan Li, Chengxin Wang

**Affiliations:** ^1^ State key Laboratory of Optoelectronic Materials and Technologies School of Materials Science and Engineering Sun Yat‐sen (Zhongshan) University Guangzhou 510275 People's Republic of China

**Keywords:** lithium–sulfur batteries, redox kinetics, single‐atom catalysts, descriptor, density functional theory

## Abstract

The sulfur redox kinetics critically matters to superior lithium–sulfur (Li–S) batteries, for which single atom catalysts (SACs) take effect on promoting Li_2_S redox process and mitigating the shuttle behavior of lithium polysulfide (LiPs). However, conventional trial‐and‐error strategy significantly slows down the development of SACs in Li–S batteries. Here, the Li_2_S oxidation processes over MN_4_@G catalysts are fully explored and energy barrier of Li_2_S decomposition (*E*_b_) is identified to correlate strongly with three parameters of energy difference between initial and final states of Li_2_S decomposition, reaction energy of Li_2_S oxidation and Li—S bond strength. These three parameters can serve as efficient descriptors by which two excellent SACs of MoN_4_@G and WN_4_@G are screened which give rise to *E*_b_ values of 0.58 and 0.55 eV, respectively, outperforming other analogues in adsorbing LiPs and accelerating the redox kinetics of Li_2_S. This method can be extended to a wider range of SACs by coupling MN_4_ moiety with heterostructures and heteroatoms beyond N where WN_4_@G/TiS_2_ heterointerface is predicted to exhibit enhanced catalytic performance for Li_2_S decomposition with *E*_b_ of 0.40 eV. This work will help accelerate the process of designing a wider range of efficient catalysts in Li–S batteries and even beyond, e.g. alkali‐ion‐Chalcogen batteries.

## Graphical abstract

Three out of nine parameters identified in this work can serve as efficient descriptors by which two excellent single atom catalysts (SACs) of MoN_4_@G and WN_4_@G with the best redox performance are screened. This descriptors‐guided design strategy can be extended to a wider range of SACs, e.g., interface and more complex coordination environments of metal center.

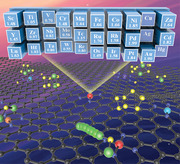

## 1 Introduction

Rechargeable lithium–sulfur (Li–S) batteries are regarded as the ideal next generation energy storage devices for their highenergy‐density and low cost.^[^ ^1^, ^2^ ^]^ The earth‐abundant sulfur cathode gives a high theoretical specific capacity of 1675 mAh g^−1^, which confers a high theoretical energy density of 2600 Wh kg^−1^ to Li–S batteries.^[^ ^3^, ^4^, ^5^ ^]^ Despite all the mentioned merits, these cells were found to be restricted to a sequence of obstacles that result in poor battery performance during cycling.^[^ ^6^ ^]^ Among them, the practical challenge named “shuttle effect” is a primary reason for the recession of Li–S batteries.^[^ ^7^ ^]^ During the discharge process, soluble long‐chain lithium polysulfide (LiPs), Li_2_S*_n_* (*n* = 4, 6, 8), can diffuse through the separator to the lithium metal anodes and be reduced to the short‐chain LiPs, Li_2_S*_n_* (*n* = 1, 2). These reduction products proliferate back to the cathode and are reoxidized to long‐chain LiPs, which leads to the irreversible capacity fading as well as low Coulombic efficiency.^[^ ^8^, ^9^, ^10^ ^]^

To address these issues and, particularly, avoid the deleterious shuttle effect, significant research efforts have been dedicated to searching for anchoring materials which are efficient in the immobilization of soluble intermediates. There are two main methodologies, namely physical confinement and chemical adsorption.^[^ ^11^, ^12^ ^]^ Although carbon materials have been integrated into cathode for hosting sulfur or LiPs, it was demonstrated that the nonpolar pristine carbon manifest weak adsorption strength to polar LiPs,^[^ ^2^ ^]^ indicating that physical confinement is far from meeting the requirement to suppress the shuttle effect. Therefore, various of materials including metal oxides,^[^ ^13^, ^14^ ^]^ sulfides,^[^ ^15^, ^16^, ^17^ ^]^ carbonaceous materials,^[^ ^18^, ^19^ ^]^ have been investigated, which exhibit stronger chemical adsorption for LiPs.^[^ ^6^, ^20^ ^]^ However, only strengthening the affinity between anchoring materials and LiPs cannot significantly suppress the shuttle effect because of the sluggish kinetics of the conversion of immobilized LiPs and Li_2_S, thereby rendering the cathode surface unexpected accumulation of LiPs and increasing the chances of LiPs diffusion toward anode. Moreover, the LiPs accumulation would cause unrestrained deposition of Li_2_S which is difficult to be fully oxidized during charge process due to high energy barrier (*E*_b_) for decomposition, which will lead to the decrease of rate capability. Therefore, increasing research attention has recently been paid to the acceleration of conversion kinetics, in which the transformation from insoluble of Li_2_S to long‐chain LiPs was highlighted.^[^ ^21^, ^22^, ^23^ ^]^

Single atom catalysts (SACs) have been proved efficient for catalyzing various of electrochemical reactions, which accordingly attracted much research attention on their applications in Li–S batteries. Up to now, MN_4_@G is the most considered model of SACs with metal coordinated by four N atoms embedded in graphene, which also inspired recent research efforts on their applications as sulfur hosts of Li–S batteries.^[^ ^24^, ^25^, ^26^ ^]^ The employment of SACs as anchoring materials can not only immobilize soluble intermediates but also accelerate redox kinetics of Li_2_S deposition.^[^ ^27^, ^28^ ^]^ However, the mechanism of Li_2_S decomposition catalyzed by SACs is far less understood and thus the key parameters affecting the catalytic performance of SACs remain unclear. Therefore, the design of SACs for Li–S batteries relies solely on the conventional trial‐and‐error method. Given numerous degrees of freedom for adjusting the local atomic environments, the catalytic properties of SACs can be tuned accordingly in a wide range,^[^ ^29^, ^30^ ^]^ which necessitate more advanced and efficient design strategy. High throughput calculations have been widely implemented to search for high efficiency SACs for various electrochemical reactions such as oxygen reduction reaction (ORR), oxygen evolution reaction (OER), hydrogen evolution reaction (HER), and N_2_ reduction reaction (NRR).^[^ ^31^, ^32^, ^33^ ^]^ Therefore, this could be an optimal strategy as well to screen SACs as high performance of anchoring materials for Li_2_S decomposition in Li–S batteries. To this regard, suitable descriptors are critical for screening high performance SACs for Li_2_S decomposition which enables exploring efficiently the configurational phase space of SACs. Although *E*_b_ of Li_2_S decomposition could be utilized as a descriptor, direct calculation of *E*_b_ consumes a large number of computational resources using the climbing‐image nudged elastic band (CI‐NEB) method,^[^ ^34^ ^]^ which hinders significantly the implementation of high throughput calculations. Therefore, to search for simple and easily obtained descriptors correlating well with *E*_b_ of Li_2_S dissociation is essential but challenging.

Herein, a series of graphene‐supported SACs as anchoring materials for Li–S batteries were systematically investigated, using density functional theory (DFT) simulations, to deepen the understanding of the mechanism underlying Li_2_S oxidation and accordingly identify the key parameters strongly correlated with the *E*_b_ of Li_2_S decomposition. The relationship of *E*_b_ and nine key parameters were extensively investigated, and we identified three of them possessing the strongest correlation with *E*_b_ which can serve as descriptors to screen SACs with excellent catalytic performance for accelerating Li_2_S oxidation from 30 systems. This descriptor‐based strategy was also extended to other catalysts containing structural features other than MN_4_ moiety.

## 2 Results

**Figure 1:**
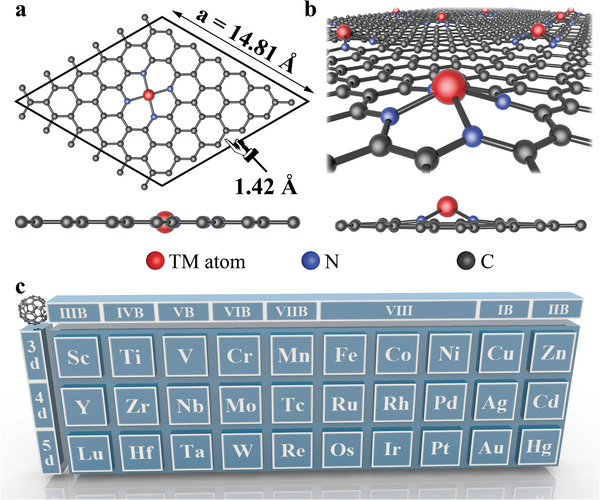
Structural features of MN_4_@G. a,b) Top and side views of the two types of relaxed structures of monolayer MN_4_@G, with the metal atom in the graphene plane or sticking out of the plane. c) Transition metal elements we considered across the periodic table, where 3*d*, 4*d*, and 5*d* represent 3*d*, 4*d*, and 5*d* transition metals, respectively.

As shown in **Figure** **1**, SACs labeled as MN_4_@G were modeled by depositing transition metal (TM) atoms onto the nitrogen doped graphene. The average bond lengths of TM—N bonds are listed in Table S1 (Supporting Information). After the geometric optimization, SACs have two type morphologies (shown in Figure 1a,b). Most of the metal atoms (V, Cr, Mn, Fe, Co, Ni, Cu, Zn, Tc, Ru, Rh, Pd, Ag, Os, Ir, Pt, and Au) prefer the in‐plane atomic arrangement, while the rest (Sc, Ti, Y, Zr, Nb, Mo, Cd, Lu, Hf, Ta, W, Re, and Hg) are somewhat different, with the TM atom sticking out of the plane.

**Figure 2:**
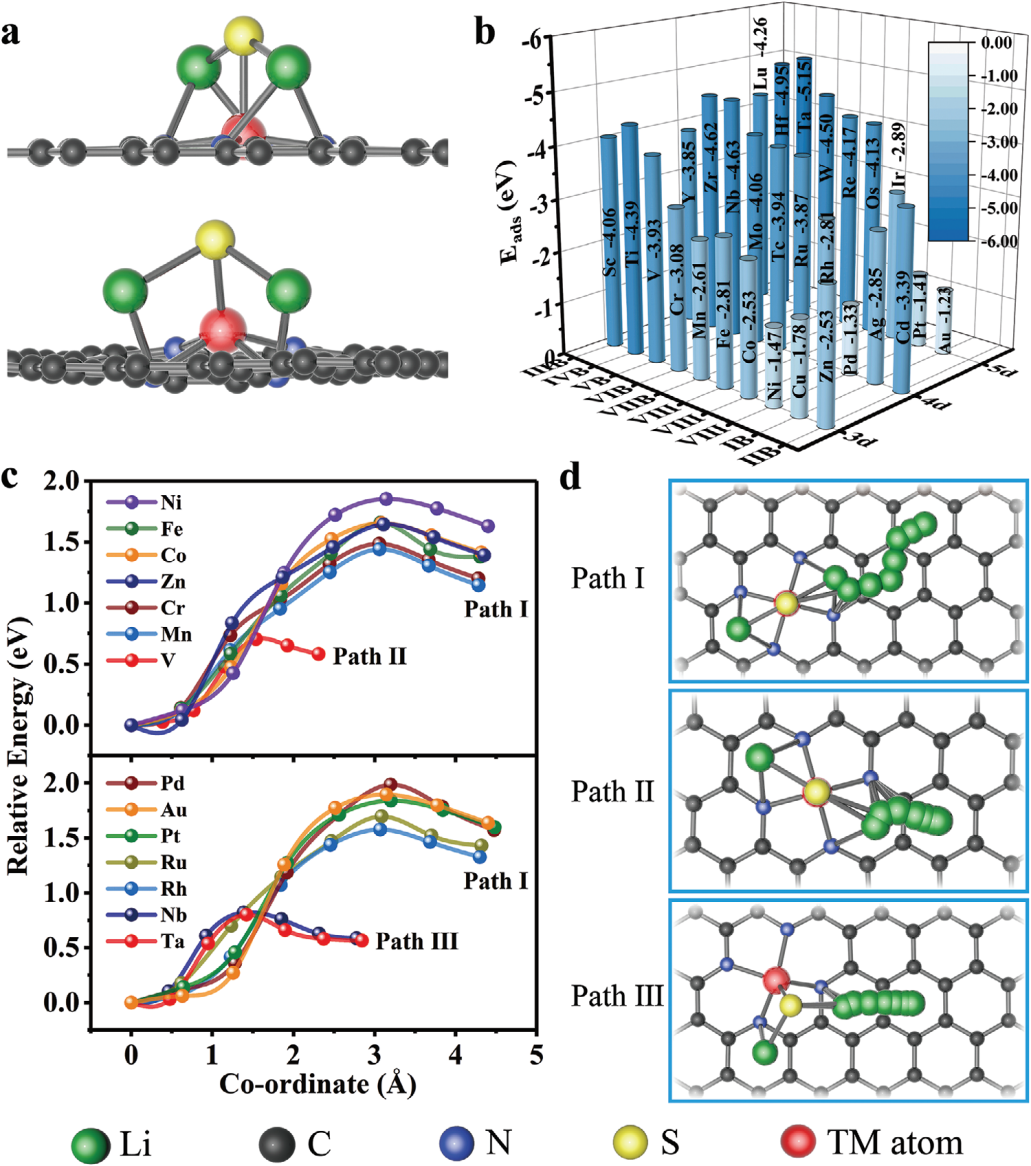
Adsorption and decomposition of Li_2_S on MN_4_@G. a) The adsorption patterns of Li_2_S on two typical SACs of VN_4_@G and TaN_4_@G, respectively. b) The adsorption energy of Li_2_S on MN_4_@G. c) The energy profiles of Li_2_S decomposition process on selected substrates. For a clear display, the element symbols represent the corresponding SACs hereafter. d) Energetically preferable Li_2_S decomposition pathways.

Various of initial adsorption configurations of Li_2_S on MN_4_@G were considered and only the most stable adsorption patterns are shown in **Figure** **2**a, where taking VN_4_@G and TaN_4_@G as examples for illustrating the Li_2_S adsorption the Li atoms are adsorbed at the hollow sites and the S atom tends to be adsorbed near the metal atom. As presented in Figure 2b and Table S2 (Supporting Information), the adsorption energies of Li_2_S are lower than −2.0 eV in most of the cases, exhibiting a stronger affinity to Li_2_S than that on graphene and consistent with the previous works.^[^ ^15^, ^35^, ^36^ ^]^ The strongest binding strength toward Li_2_S was found on TaN_4_@G, with the *E*_ads_ value of −5.15 eV and the weakest interaction took place on AuN_4_@G (*E*_ads_ = −1.23 eV). Group IIIB, IVB, VB, and VIB elements exhibit ultrastrong adsorption strength to Li_2_S (*E*_ads_ are all less than −3.0 eV), while VIII and IB elements show poor adsorption properties. The *E*_ads_ of Li_2_S on VN_4_@G, MnN_4_@G, FeN_4_@G, CoN_4_@G, NiN_4_@G, and ZnN_4_@G are −3.93, −2.61, −2.81, −2.53, −1.47, and −2.53 eV, respectively, which is in good agreement with the previous report.^[^ ^36^ ^]^ The substrates of AgN_4_@G with Li_2_S adsorption and HgN_4_@G show significant structural distortions (as shown in Figure S1a,b in the Supporting Information). In this concern those two substrates are not studied further.

The reaction kinetics during charging process is dominated by the Li_2_S decomposition.^[^ ^8^ ^]^ We therefore investigated the catalytic performance of fourteen randomly selected SACs in Li_2_S oxidation by calculating the decomposition barriers using CI‐NEB method. The energy profiles are shown in Figure 2c and the energetically optimal reaction pathways for Li_2_S decomposition are shown in Figure 2d, where three kinds of paths are identified based on their lengths. The decomposition of Li_2_S on VN_4_@G follows the manner of path II, on NbN_4_@G and TaN_4_@G path III, and on the other substrates path I. The adsorption state Li_2_S undergoes Li—S bond breaking process to form LiS cluster and a single Li ion. It can be clearly seen that VN_4_@G, TaN_4_@G and NbN_4_@G shows the best catalytic properties with the *E*_b_ values of 0.70, 0.80, and 0.82 eV, respectively, and in comparison, the greatest *E*_b_ value that reaches to 1.99 eV was found on PdN_4_@G. It should be noted that Li_2_S on VN_4_@G undergoes an energetically more favorable decomposition process compared to that reported in the literature,^[^ ^35^ ^]^ which is attributed to its shorter decomposition pathway. Li_2_S decomposition barriers on various SACs including CrN_4_@G, MnN_4_@G, FeN_4_@G, CoN_4_@G, NiN_4_@G, ZnN_4_@G, RuN_4_@G, RhN_4_@G, PtN_4_@G, and AuN_4_@G are 1.48, 1.44, 1.66, 1.66, 1.85, 1.64, 1.70, 1.58, 1.84, and 1.90 eV in the given order. These results are consistent with the previous studies.^[^ ^35^ ^]^

**Figure 3:**
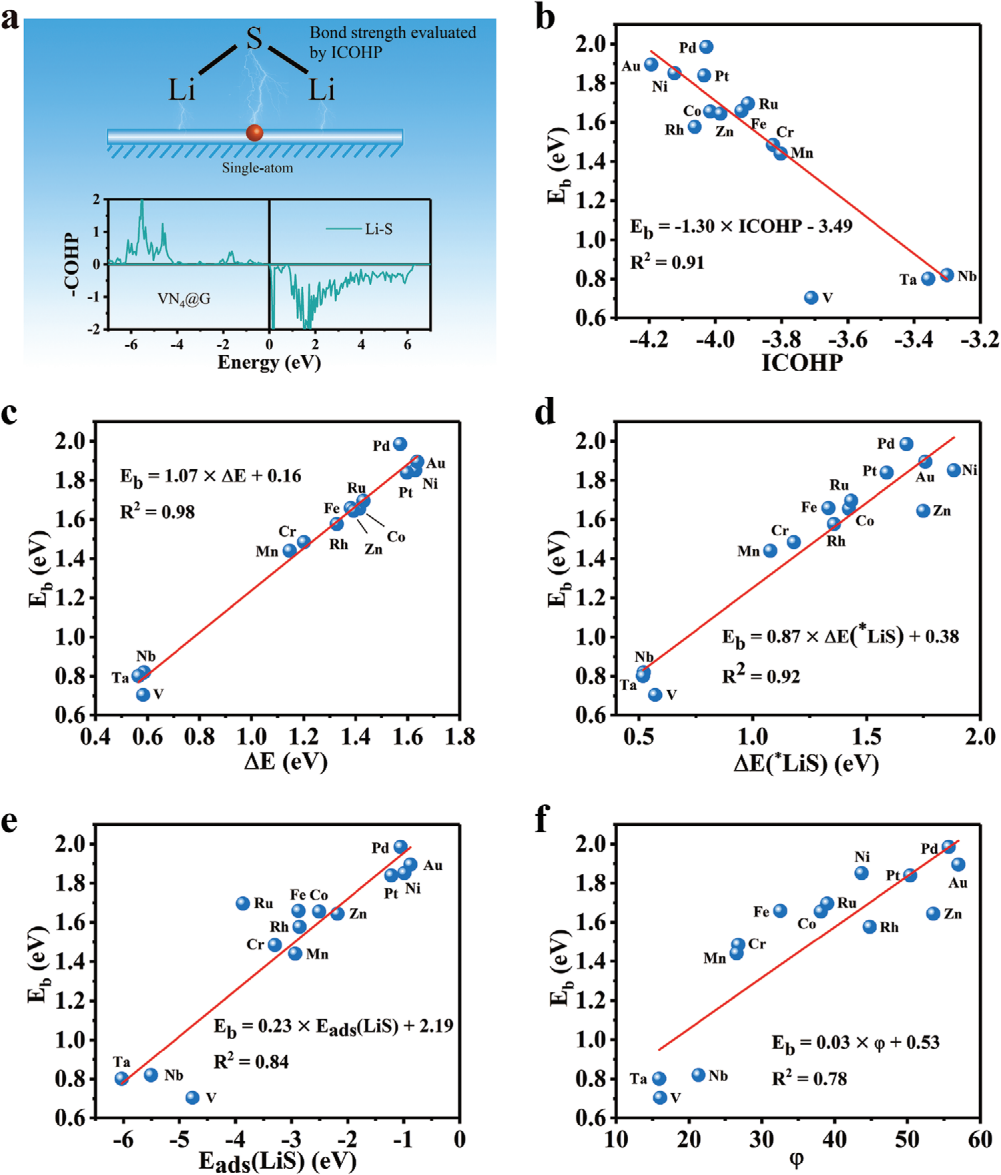
Potential descriptors for Li_2_S decomposition. a) Schematic diagram for the mechanism of Li_2_S decomposition and COHP for VN_4_@G. Scatters of *E*_b_ versus ICOHP of Li–S interaction b), Δ*E* c), Δ*E*(*LiS) d), *E*_ads_ (LiS) e), and *φ* f), respectively. The red lines in b–f) represent the corresponding linear relationships. The correlation functions and *R* ^2^ values are also given.

To dig more deeply into the mechanism of Li_2_S decomposition, it is straightforward to examine the intensity of Li–S interaction after the Li_2_S adsorption as illustrated in **Figure** **3**a. Hence, Crystal Orbital Hamilton Population (COHP) analysis was introduced to evaluate the interaction of Li and S atoms as Li_2_S was trapped by the SACs^[^ ^37^, ^38^ ^]^ and the “bond energies” can be characterized by the interatomic COHP values.^[^ ^39^ ^]^ Therefore, the integrated values of COHP (ICOHP), calculated by the corresponding energy integral below Fermi level (*E*_f_) (Figure 3a), were used to quantitatively explore the bonding strength of Li–S bond. The smaller value of ICOHP represents a stronger interatomic interaction and vice versa. As can be seen in Figure 3b, the desirable linear correlation between these two variables is evidenced, which is proven by the good coefficient of determination value (*R* ^2^) of 0.91. The ICOHP values of Li–S interaction on VN_4_@G, NbN_4_@G, and TaN_4_@G surfaces are −3.71, −3.30, and −3.36, which are larger than those for their analogues demonstrating weaker chemical bonds of Li–S and lower *E*_b_ values in the former three SACs. Note that, the data point for VN_4_@G is a statistical outlier because it possesses lower ICOHP than that for NbN_4_@G and TaN_4_@G but gives rise to smaller *E*_b_. This discrepancy could be attributed to the additional substrate affinity to Li atoms (see Figure S2, Supporting Information). As listed in Table S3 (Supporting Information), in VN_4_@G the lowest average values of ICOHP of Li–N and Li–C are around −1.11 and −0.71, lower than those for TaN_4_@G and NbN_4_@G, which means a stronger binding strength between Li and VN_4_@G facilitating the decomposition of Li_2_S.^[^ ^40^ ^]^ The structure of Li_2_S adsorbed on NiN_4_@G and AuN_4_@G are the least to be decomposed, reflected by the most negative ICOHP values of −4.12 and −4.19 up to *E*_f_ severally. The quantitative bond strength for other cases can be found in Figure S3 (Supporting Information). These results indicate that the ease of Li_2_S decomposition is dominated by the Li—S bond strength in the absorbed phase of Li_2_S.

We next investigated several simple parameters and explored their correlations with *E*_b_. We considered the energy difference of initial and final states of Li_2_S decomposition (Δ*E*) essential for calculating *E*_b_ based on CI‐NEB method, which can be defined as: Δ*E* = *E*(*Li+*LiS) − *E*(*Li_2_S), where *E*(*Li+*LiS) and *E*(*Li_2_S) represent the energies of final and initial states as illustrated in Figure 2d. The adsorbates with an asterisk “*” on the upper left refers to the adsorption state. As shown in Figure 3c, the fitted equation can be described as: *E*_b_ = 1.07 × Δ*E* + 0.16 (Δ*E* > 0 eV), with the remarkable *R* ^2^ of 0.98, signaling that there is an extremely strong positive correlation between Δ*E* and *E*_b_. To this end, Δ*E* could be used as an effective adsorption descriptor, with which we can precisely estimate the *E*_b_ values for evaluating the catalytic performance of SACs toward Li_2_S oxidation.

To a significant degree the catalytic performance of a metal is determined by the affinity strength of the reaction intermediates of Li_2_S decomposition on the surface of the catalyst.^[^ ^41^ ^]^ As the first step of charging process in Li–S batteries, decomposition of Li_2_S could also be described by *Li_2_S → *LiS + Li^+^ + e^−^ where Li^+^ is isolated in vacuum instead of being adsorbed on SACs, which is different from the process aforementioned for calculating *E*_b_ based on CI‐NEB method. To calculate reaction energy is a prevailing method to evaluate the performance of catalysts for electrochemical reactions. Thereby we here introduced reaction energy Δ*E*(*LiS) = *E*(*LiS) + *E*(Li)_bcc_ − *E*(*Li_2_S) as a possible adsorption descriptor for Li_2_S oxidation, where *E*(*LiS), *E*(Li)_bcc_, *E*(*Li_2_S) refer to the total energy of adsorbed LiS cluster, energy of single Li atom in bcc bulk phase and total energy of adsorbed Li_2_S, respectively. Using the scaling relationships, *E*_b_ as a function of Δ*E*(*LiS) was plotted in Figure 3d which could be described by the equation of *E*_b_ = 0.87 × Δ*E*(*LiS) + 0.38 with *R* ^2^ = 0.92.

Inspired by the previous works of water splitting^[^ ^42^ ^]^ and CO_2_ reduction,^[^ ^43^ ^]^ we explored the possibility of adsorption energy of LiS (*E*_ads_ (LiS)) serving as an additional descriptor for Li_2_S oxidation. Using the scaling relations, *E*_b_ as a function of *E*_ads_ (LiS) was investigated (*R* ^2^ = 0.84, illustrated in Figure 3e). Although there is a relatively large standard deviation appears for the data of *E*_ads_ (LiS), it still resonates roughly with the results from ICOHP, Δ*E*, and *E*(*LiS) (Figure 3b–d) in particular for those VN_4_@G, TaN_4_@G, and NbN_4_@G.

Recently, a descriptor *φ* having all parameters related to intrinsic properties of catalytic center,^[^ ^31^ ^]^ which can be expressed as φ = *θ* × (*χ*_M_ + *αn*_N_ *χ*_*N*_)/*χ*_S_, containing the information of valence electrons in the occupied *d* orbit of TM atom (*θ*) and the electronegativity of its ligand atoms (*χ*) has been proposed to design electrocatalysts toward ORR, OER, and HER.^[^ ^32^, ^44^ ^]^ We set up *φ* in the same way, where *a* is set to 1, and *n*_N_ *_,_ χ*_N_, and *χ*_S_ are the number of nearest‐neighbor nitrogen atoms, electronegativity of nitrogen and sulfur, respectively. Its correlation with *E*_b_ was explored. As can be seen from Figure 3f that the linear relationship between *φ* and *E*_b_ is not so remarkable as represented by a low *R* ^2^ value of 0.78. This might be caused by the fact that *φ* does not precisely describe the intrinsic properties of SACs. As we found that *φ* is greatly determined by the periodic law, reflected by *θ* and *χ*_M_, and the electronegativity of N has a very limited modification to the goodness of fit of *φ*‐*E*_b_ relationship (see Figure S4, Supporting Information).

We also explored the correlation of *E*_b_ and intrinsic properties of adsorbed phase of Li_2_S, e.g., the changes of bond length, bond angle, charge of Li_2_S, and charge of Li atom. Upon the deposition of Li_2_S, the larger the variations in bond length and bond angle of Li_2_S are, the weaker of the bonding strength of Li—S bond is.^[^ ^45^ ^]^ The variation behaviors of bond length and bond angle with respect to elements coincide roughly with that for *E*_b_, with correlation coefficients of 0.83 and 0.43, respectively. It can be seen from Figure S5a,b (Supporting Information) that the largest elongation of bond length is observed on VN_4_@G, TaN_4_@G, and NbN_4_@G, reaching 0.29, 0.37, and 0.36 Å apiece, and the greatest variation in bond angle also occurred on TaN_4_@G and NbN_4_@G substrates, reaching absolute values of 21.35° and 25.37°, which are much larger than that on VN_4_@G (16.18°). These results generally indicate that VN_4_@G, TaN_4_@G, and NbN_4_@G substrates could weaken the bonding of Li and S atoms, which is known as adsorption activation.^[^ ^21^ ^]^ In contrast, Li_2_S adhere to NiN_4_@G, PdN_4_@G, PtN_4_@G, and AuN_4_@G can maintain the structure similar to the pristine one indicating their worse catalytic performance than TaN_4_@G and NbN_4_@G for the Li_2_S decomposition. More details of bond length and bond angles of adsorbed Li_2_S are shown in Table S4 (Supporting Information). Moreover, Bader charge analysis^[^ ^46^ ^]^ was performed to investigate the adsorption induced charge variations of Li_2_S (Figure S5c, *R* ^2^ = 0.12, Supporting Information) and Li atom (Figure S5d, *R* ^2^ = 0.76, Supporting Information), respectively. In particular, the adsorption induced charge difference of Li in Li_2_S varies roughly linearly with respect to *E*_b_, indicating that the activation of Li atom is more beneficial to the decomposition of Li_2_S.

Overall, based on the 14 randomly selected systems of SACs we gained in‐depth understanding the mechanism of Li_2_S decomposition and extracted the relationships of *E*_b_ and nine parameters (Figure 3; and Figure S5, Supporting Information). Among them, Δ*E*, Δ*E*(*LiS), and ICOHP can better correlate with *E*_b_ than *E*_ads_ (LiS), *φ*, and other parameters evidencing by their better goodness of fit, which could serve as descriptors for fast screening.

**Figure 4:**
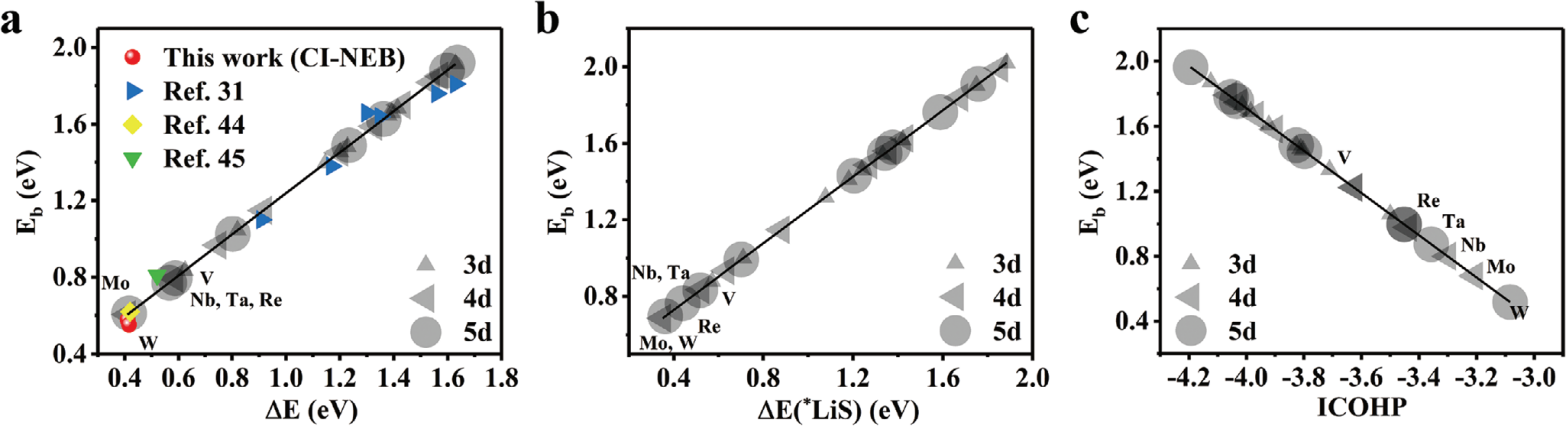
Prediction and screening of efficient catalysts among MN_4_@G. Prediction and screening for active SACs based on Δ*E* a), Δ*E*(*LiS) b), and ICOHP c), where the gray symbols represent the predicted results and colorful symbols in a) represent the CI‐NEB results reported in this work or previous works. The black lines are the correlation functions obtained in previous discussion.

We focus mainly on three descriptors of Δ*E*, Δ*E*(*LiS), and ICOHP to explore their predictive capability for estimating *E*_b_ and further to screen high performance SACs for Li_2_S decomposition. Before that we first validated the prediction capability of Δ*E* using the available data from the literature about the decomposition barriers of Li_2_S over other SACs anchored on N doped graphene and pristine C_2_N.^[^ ^35^, ^47^, ^48^ ^]^ As shown in **Figure** **4**a, the data points all fall near the predicted line, indicating the robustness of Δ*E*−*E*_b_ linear relationship. This gives us confidence that this descriptor of Δ*E* can provide efficient theoretical guidance for fast screening active SACs for Li–S batteries. Thus, the descriptor of Δ*E* was utilized to evaluate the catalytic performance of all SACs systems by calculating *E*_b_ values based on the equation of *E*_b_ = 1.07 × Δ*E* + 0.16 (Δ*E* > 0) as shown in Figure 4a.

As can be seen from Figure 4a, those SACs with significantly low decomposition barriers (*E*_b_ < 0.85 eV) are found to be WN_4_@G, MoN_4_@G, NbN_4_@G, TaN_4_@G, VN_4_@G, and ReN_4_@G which comprise middle transition metal atoms having both partially occupied *d* orbitals and enough *d* electrons. In particular, SACs of Mo and W on N_4_@G are predicted to be the potential catalysts with the highest catalytic activity, which even have lower *E*_b_ than VN_4_@G benchmark.^[^ ^35^ ^]^ This means that among 30 SACs MoN_4_@G and WN_4_@G could catalyze Li_2_S oxidation with the fastest kinetics. Their low *E*_b_ values could also be reflected by the variation in bond length and the Bader charge of Li atom upon adsorption where the most significant change occurs for Li_2_S on WN_4_@G and MoN_4_@G, as shown in Figure S6 (Supporting Information). Again, the prediction power of these three descriptors was validated by the *E*_b_ for WN_4_@G, MoN_4_@G directly calculated using CI‐NEB method (Figure 4; and Figure S7, Supporting Information). Amazingly, the CI‐NEB method obtained *E*_b_ values of 0.55 and 0.58 eV are nicely consistent with the results of 0.61 and 0.61 eV given by Δ*E*‐*E*_b_ relationship. Note that the final state of Li_2_S disassociation on CuN_4_@G suffers from lattice deformation (Figure S1c, Supporting Information), and hence CuN_4_@G was not furtherly studied.

Δ*E*(*LiS) and ICOHP could reproduce the trend of *E*_b_, as shown in Figure 4b,c, generated by Δ*E* indicating they are also good descriptors for screening SACs although the *E*_b_ values predicted by ICOHP and Δ*E*(*LiS) have bigger discrepancies than by Δ*E* with respect to those given by CI‐NEB method as listed in Table S5 (Supporting Information). The only exception happens to VN_4_@G predicted by ICOHP, which has been aforementioned that this big discrepancy is assigned to the relatively strong interaction between Li atoms and substrate. Otherwise, the predictive results of *E*_ads_(LiS), *φ* and other parameters related to adsorbed Li_2_S are significantly different from those predicted by Δ*E*, Δ*E*(*LiS), and ICOHP as illustrated in Figure S8, Tables S5, and S6 (Supporting Information), which demonstrate their worse predictive capability due to their weaker correlation with *E*_b_.

Based on the equations of *E*_b_ = 1.07 × Δ*E* + 0.16 (Δ*E* > 0), *E*_b_ = 0.87 × Δ*E*(*LiS) + 0.38, and *E*_b_ = −1.30 × ICOHP −3.49, one can expect SACs with MN_4_ moiety possessing better catalytic performance than WN_4_@G and MoN_4_@G for catalyzing Li_2_S oxidation should have Δ*E* < 0.36 eV, Δ*E*(*LiS) < 2.22 eV, or ICOHP > −3.11. This needs further investigations.

**Figure 5:**
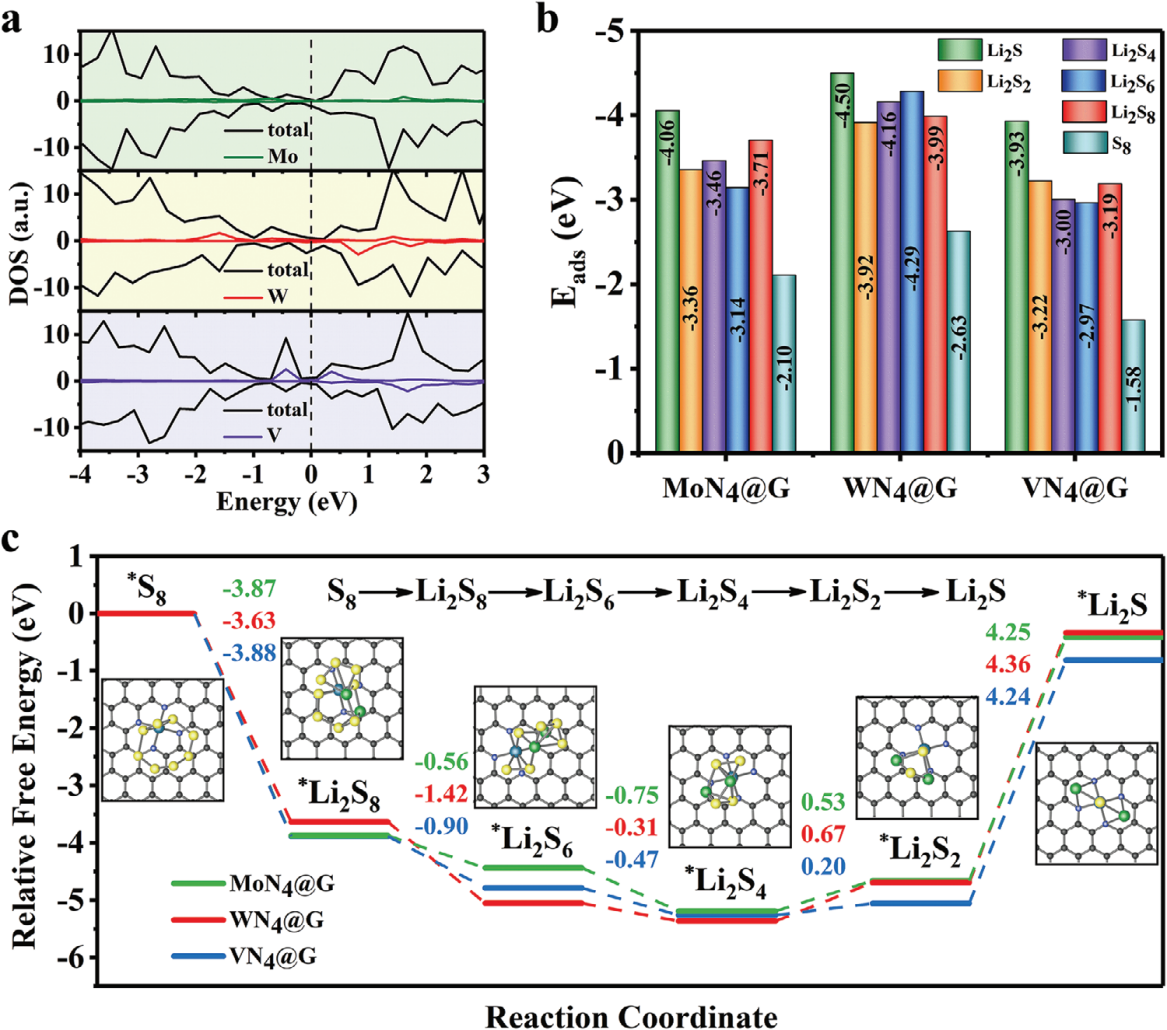
Electrochemical, adsorption, and reduction performances of the screened SACs. a) The DOS of MoN_4_@G, WN_4_@G, and VN_4_@G, where the Fermi level (the vertical dashed line) was set to zero. b) The adsorption energies of different lithiation states on MoN_4_@G, WN_4_@G, and VN_4_@G. The corresponding *E*_ads_ values were labeled on the bars. c) Energy profiles for the reduction of sulfur on MoN_4_@G, WN_4_@G, and VN_4_@G. The atomic structures are typical adsorption conformations of S_8_ and LiPs species on the screened SACs and the benchmark.

Before further investigating the performances of MoN_4_@G and WN_4_@G as anchoring materials in Li–S batteries, we then explored their stability to clarify the experimental feasibility of synthesis. We found that these three SACs are more energetically stable embedded in the center of N_4_@G moiety than anchored on the nearby graphene sites, which is shown in Figure S9 (Supporting Information), indicating the feasibility for experimental synthesis. Moreover, the density of states (DOS) of MoN_4_@G, WN_4_@G, and VN_4_@G were investigated in **Figure** **5**a, which reveals that these substrates are electrical conductors without bandgap. Note that VN_4_@G was set as a benchmark in this study, which was treated as the optimum catalyst in previous work.^[^ ^35^ ^]^

DFT calculations were then performed to examine the adsorption performance of those catalysts for all of the reaction intermediates during the redox of sulfur. As demonstrated in Figure 5b, the *E*_ads_ of LiPs on MoN_4_@G, WN_4_@G, and VN_4_@G are almost less than −3.0 eV, showing strong adsorption strength. Taking the adsorption of Li_2_S_6_ as an example, the *E*_ads_ are −3.14, −4.29, and −2.96 eV on MoN_4_@G, WN_4_@G, and VN_4_@G, respectively. Previous work demonstrates that strong affinity strength to LiPs can effectively mitigate the shuttle effect.^[^ ^15^ ^]^ It can be seen that the *E*_ads_ of all intermediates on MoN_4_@G and WN_4_@G are lower than that on VN_4_@G benchmark, exhibiting a stronger adsorption strength, which suggests MoN_4_@G and WN_4_@G are potential optimum anchoring materials for entrapping LiPs.

To gain further insights into the kinetics of lithiation process, the Gibbs free energy profiles for discharge reactions from S_8_ to Li_2_S were calculated and exhibited in Figure 5c. The entire reversible reaction is complex and the reaction from Li_2_S_8_ to Li_2_S is accompanied by the production of isolated state Li_2_S_2_ (Equation (3)–(8) in the Supporting Information).^[^ ^49^ ^]^ The overall free energy changes are −0.41, −0.33, and −0.82 eV for MoN_4_@G, WN_4_@G, and VN_4_@G, respectively, suggesting that the overall reaction on those substrates are exothermic. Whereas, for individual lithiation step, the conversion from S_8_ to Li_2_S_4_ are exothermic and the subsequent two steps are endothermic. Obviously, the reduction from Li_2_S_2_ to Li_2_S possess the largest positive change of Gibbs free energy, indicating that the conversion from Li_2_S_2_ to Li_2_S is the rate‐determining step (RDS) in the entire lithiation process. The reduction kinetics can be characterized by the change of Gibbs free energy of RDS (Δ*G*_RDS_). As can be seen from Figure 5c, the lithiation process of sulfur on MoN_4_@G (Δ*G*_RDS_ = 4.25 eV) and WN_4_@G (Δ*G*_RDS_ = 4.36 eV) exhibiting similar efficiency as that on VN_4_@G benchmark (Δ*G*_RDS_ = 4.24 eV). Given the excellent catalytic performance for Li_2_S oxidation, WN_4_@G and MoN_4_@G exhibit remarkable performance of adsorbing LiPs, accelerating reduction kinetics of Li_2_S, which will significantly suppress the shuttle effect and improve the rate performance of Li–S batteries and in particular will be expected to perform better than ever reported VN_4_@G. This deserves further experimental investigations.

Conversion of LiPs and Li_2_S is critical for suppressing shuttle effect and thus a superior catalyst should keep balance between both oxidation and reduction processes. To confirm the overall catalytic performances of those screened VN_4_@G, MoN_4_@G, and WN_4_@G, we further explored the redox processes of other MN_4_@G (M = Ti, Zr, Nb, Tc, Hf, Ta, and Re) which possess Δ*E* < 1.0 eV. As shown in Figures S10 and S11 (Supporting Information), the free energy diagrams for reduction process over those SACs demonstrate that the RDS is the Li_2_S_2_ → Li_2_S, consistent with previous work.^[^ ^24^, ^49^, ^50^ ^]^ When Δ*E* < 1.0 eV, we now have 10 SACs whose performances are compared based on the values of Δ*G*_RDS_ and *E*_b_ summarized in Table S7 (Supporting Information). Generally, *E*_b_ undergoes significant change by 108% from WN_4_@G (*E*_b_ = 0.55 eV) to TcN_4_@G (*E*_b_ = 1.15 eV) indicating a pronounced diversity of catalytic performances of those SACs, while Δ*G*_RDS_ varies from ZrN_4_@G (Δ*G*_RDS_ = 3.83 eV) to TaN_4_@G (Δ*G*_RDS_ = 5.36 eV) by 40% maximum. Except for Ta, other SACs exhibit comparable capability for reduction process due to similar values of Δ*G*_RDS_. We found SACs (Mo/W/Re/NbN_4_@G) are as active as VN_4_@G^[^ ^35^ ^]^ for both reduction and oxidation of Li_2_S. Among them, MoN_4_@G and WN_4_@G with superior activity for Li_2_S oxidization deserve further investigation in experiment.

**Figure 6:**
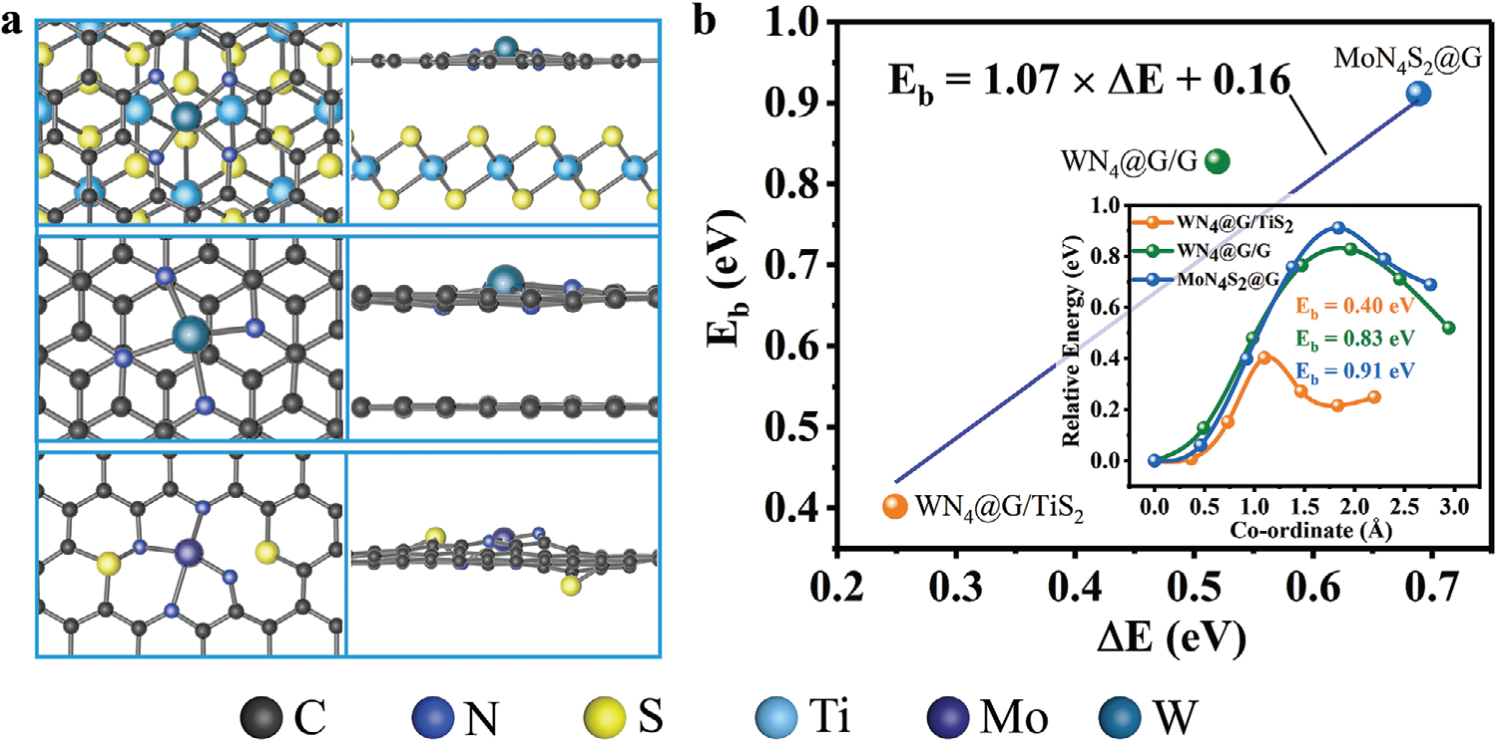
Extension of Δ*E* to SACs beyond MN_4_. a) The optimized atomic configurations of WN_4_@G/TiS_2_(top), WN_4_@G/G (middle), and MoN_4_S_2_@G(bottom). b) Comparison of *E*_b_ given by CI‐NEB method and *E*_b_‐Δ*E* linear relationship for the three substrates, respectively, where CI‐NEB obtained *E*_b_ values for WN_4_@G/TiS_2_, WN_4_@G/G, and MoN_4_S_2_@G are represented by orange, green and blue dots, respectively. The insert graph is the energy profiles for Li_2_S decomposition process.

We revealed that Δ*E*, Δ*E*(*LiS), and ICOHP can serve as distinguished descriptors for rationally designing potential catalysts of MN_4_@G catalyzing Li_2_S decomposition and next we extended this strategy to predict the catalytic performance of various types of 2D materials with dispersed atomic metals for Li_2_S decomposition. Recently, various strategies have been proposed to enhance the catalytic performances of SACs toward electrochemical reactions, which involve heterostructures^[^ ^51^, ^52^, ^53^ ^]^ and heteroatoms dopants other than N to tailor the local atomic environments.^[^ ^54^ ^]^ Therefore, we used Δ*E*, Δ*E*(*LiS), and ICOHP to search for enhanced catalytic performance compared to WN_4_@G and MoN_4_@G by introducing interface effect and S dopant to modulate electronic properties of active metal center.^[^ ^55^, ^56^ ^]^ We constructed three typical models as representatives shown in **Figure** **6**a including two heterostructures (WN_4_@G/TiS_2_ and WN_4_@G/G) and one MN_4_@G with tailored local environment (MoN_4_S_2_@G). WN_4_@G/TiS_2_ and WN_4_@G/G are built by introducing monolayer of TiS_2_ and graphene, respectively, while MoN_4_S_2_@G is realized by doping two sulfur atoms into the second coordination sphere of TM atom. Based on the fitted equation of *E*_b_ = 1.07 × Δ*E* + 0.16, the estimated *E*_b_ values of Li_2_S for WN_4_@G/TiS_2_, WN_4_@G/G, and MoN_4_S_2_@G are 0.43, 0.72, and 0.90 eV, respectively. Amazingly, additional CI‐NEB calculations give rise to *E*_b_ of 0.40, 0.83, and 0.91 eV in the given order, confirming the prediction of descriptor Δ*E*, as shown in Figure 6b. Whereas the ICOHP of Li–S interaction reproduced the trend but relatively large *E*_b_ values (**Table** **1**). On the contrary, based on Δ*E*(*LiS) one can obtain the largest deviation with those from CI‐NEB method in particular for the case of WN_4_@G/TiS_2_. Moreover, the trend of *E*_b_ given by CI‐NEB method could not be reproduced by Δ*E*(*LiS). Therefore, Δ*E*(*LiS)‐*E*_b_ relationship needs to be modified for specific ligand environment which needs further investigation.

**Table 1:** Predictive capability of descriptors for SACs beyond MN_4_. The *E*_b_ values of Li_2_S decomposition for the extension systems given by Δ*E*, ICOHP, Δ*E*(*LiS), and CI‐NEB method. All data are in units of eV

SACs	*E*_b_ [ΔE]	*E*_b_ [ICOHP]	*E*_b_ [*LiS]	*E*_b_ [CI‐NEB]
WN_4_@G/TiS_2_	0.43	0.55	0.92	0.40
WN_4_@G/G	0.72	0.96	0.79	0.83
MoN_4_S_2_@G	0.90	1.02	1.15	0.91

Heterostructure of WN_4_@G/TiS_2_ possesses the merits of its individual components which will behave better than TiS_2_ or WN_4_@G in suppressing shuttle effect since WN_4_@G/TiS_2_ shows superior catalytic performance outperforming WN_4_@G monolayer. All these results not only evidence that the incorporation of monolayer TiS_2_ could further reduce the energy barrier of Li_2_S decomposition over WN_4_@G but also confirm the rationality that Δ*E* and ICOHP can be extended to wider range of catalysts for Li_2_S oxidization. Generally, these two parameters could guide further investigations for rationally designing SACs with heterostructures and/or tailored MN*_x_* moiety. Any other catalysts having configurations like MN_4_@G/TiS_2_ and MN_4_S_2_@G are expected to have better catalytic performance for Li_2_S decomposition when their Δ*E* < 0.36 eV or ICOHP > −3.11. In addition, we believe this screening strategy could also be extended to screen catalyst in alkali‐ion‐Chalcogen batteries.^[^ ^57^, ^58^ ^]^

## 3 Conclusion

In summary, we systematically investigated the mechanism of Li_2_S oxidation catalyzed by graphene supported SACs and identified three out of nine parameters correlating strongly with the energy barrier (*E*_b_) of Li_2_S dissociation, which are bond strength (evaluated by ICOHP), energy difference of initial and final states of Li_2_S decomposition (Δ*E*) and reaction energy of Li_2_S decomposition (*E*(*LiS)). The equations describing the relationships of *E*_b_, Δ*E*, and ICOHP are *E*_b_ = 1.07 × Δ*E* + 0.16 (Δ*E* > 0), *E*_b_ = 0.87 × Δ*E*(*LiS) + 0.38, and *E*_b_ = −1.30 × ICOHP −3.49, respectively. Under the guidance of descriptors Δ*E*, Δ*E*(*LiS), and ICOHP, MoN_4_@G, and WN_4_@G were screened out as optimal catalysts for catalyzing Li_2_S oxidation with the *E*_b_ values of 0.58 and 0.55 eV, as well as competitive trapping capability and reduction activity, indicating they can serve as superior performance anchoring materials in Li–S batteries. More importantly, the efficient descriptors Δ*E* and ICOHP could be extended to predict the decomposition barrier of Li_2_S on various types of catalysts containing dispersed TM atom centers, such as heterointerface and SACs of MN_4_@G with more complex local environment. Heterointerface of WN_4_@G/TiS_2_ exhibit promising catalytic performance for Li_2_S decomposition outperforming WN_4_@G by giving rise to *E*_b_ of 0.40 eV. We believe that these efficient descriptors of Δ*E*, *E*(*LiS), and ICOHP could help in fast screening and designing wider range of electrochemical catalysts in Li–S batteries and even other alkali‐ion‐Chalcogen batteries.

## Conflict of Interest

The authors declare no conflict of interest.

## Supporting information

Supporting information

## Data Availability

Research data are not shared.
