# Supplementary material for: Universal‐Descriptors‐Guided Design of Single Atom Catalysts toward Oxidation of Li2S in Lithium–Sulfur Batteries
Source: Adv Sci (Weinh). 2021 Oct 20;8(23):2102809. doi: 10.1002/advs.202102809 (PMC8655168; doi:10.1002/advs.202102809)
Supplement: Supplementary file 1 — Supporting information [file ADVS-8-2102809-s001.pdf]

ADVANCED SCIENCE
Open Access

Supporting Information

for *Adv. Sci.*, DOI: 10.1002/advs.202102809

Universal-Descriptors-Guided Design of Single Atom Catalysts toward Oxidation of Li_2S in Lithium-Sulfur Batteries

Zhihao Zeng, Wei Nong, Yan Li, Chengxin Wang**

Supporting Information

Universal-Descriptors-Guided Design of Single Atom Catalysts toward Oxidation of Li_2S in Lithium-Sulfur Batteries

Zhihao Zeng, Wei Nong, Yan Li*, Chengxin Wang*

State key Laboratory of Optoelectronic Materials and Technologies, School of Materials Science and Engineering, Sun Yat-sen (Zhongshan) University, Guangzhou 510275, People's Republic of China

* Corresponding Authors

E-mail: liyan266@mail.sysu.edu.cn; wchengx@mail.sysu.edu.cn

Computational Details: All of the first-principle calculations were performed taking the spin polarization into consideration based on the density functional theory (DFT), which was implemented in the Vienna ab initio simulation package (VASP).[1] The projector-augmented wave (PAW) method was applied to express the electron-ion interaction,[2] and the generalized gradient approximation (GGA) exchange-correlation function were profiled by the Perdew-Burke-Ernzerhof (PBE).[3] Additionally, we used the DFT-D2 of van der Waals (vdWs) corrections in the simulation.[4] The model of SACs was built by depositing MN_4 moiety into $6 \times 6 \times 1$ supercell of graphene, which contains 71 atoms. The vacuum thickness is set to 20 Å along the *z*-direction for all supercells to avoid periodic interactions. The kinetic energy cut-off for the plane-wave basis expansion is set to 520 eV. The integration of Brillouin zone was performed using a *k*-point mesh of $2 \times 2 \times 1$ Gamma-center Monkhorst-Pack for geometric optimization and $3 \times 3 \times 1$ Gamma-center Monkhorst-Pack for static self-consistent field (SCF) calculation.[5] A denser mesh of $17 \times 17 \times 1$ was used to calculate the density of states (DOS). To gain insights into the reaction kinetics on the surface of different substrates, the CI-NEB method was applied to calculate the energy barriers for Li_2S decomposition.

The adsorption energy (E_{ads}) of adsorbates were calculated according to

$$E_{ads} = E(\text{total}) - E(\text{substrate}) - E(\text{adsorbate}) \quad (1)$$

where E(total), E(substrate) and E(adsorbate) represent the energies for adsorbed systems, MN_4@G substrates, and isolated adsorbates, respectively. According to this definition, the greater the negative value of E_{ads}, the stronger the interaction between the adsorbates and substrates.

The Gibbs free energy of Li_2S_n and S_8 are calculated by

$$G = E_{DFT} + E_{ZPE} - TS \quad (2)$$

where E_{DFT}, E_{ZPE}, and S stand for the total energy obtained by DFT simulation, zero-point energy, and the entropy, respectively.

The elementary reaction steps for sulfur reduction[6] are considered as:

$$S_8 + 16Li^+ \rightarrow S_8^* + 16Li^+ \#(3)$$

$$S_8^* + 16Li^+ + 2e^- \rightarrow Li_2S_8^* + 14Li^+ \#(4)$$

$$Li_2S_8^* + 14Li^+ + 2e^- \rightarrow Li_2S_6^* + Li_2S_2 + 12Li^+ \#(5)$$

$$Li_2S_6^* + Li_2S_2 + 12Li^+ + 2e^- \rightarrow Li_2S_4^* + 2Li_2S_2 + 10Li^+ \#(6)$$

$$Li_2S_4^* + 2Li_2S_2 + 10Li^+ + 2e^- \rightarrow Li_2S_2^* + 3Li_2S_2 + 8Li^+ \#(7)$$

$$Li_2S_2^* + 3Li_2S_2 + 8Li^+ + 8e^- \rightarrow Li_2S^* + 7Li_2S\#(8)$$

The energy of a single Li ion and an electron (Li^+ + e^-) pair was treated as the energy of a crystalline Li atom.

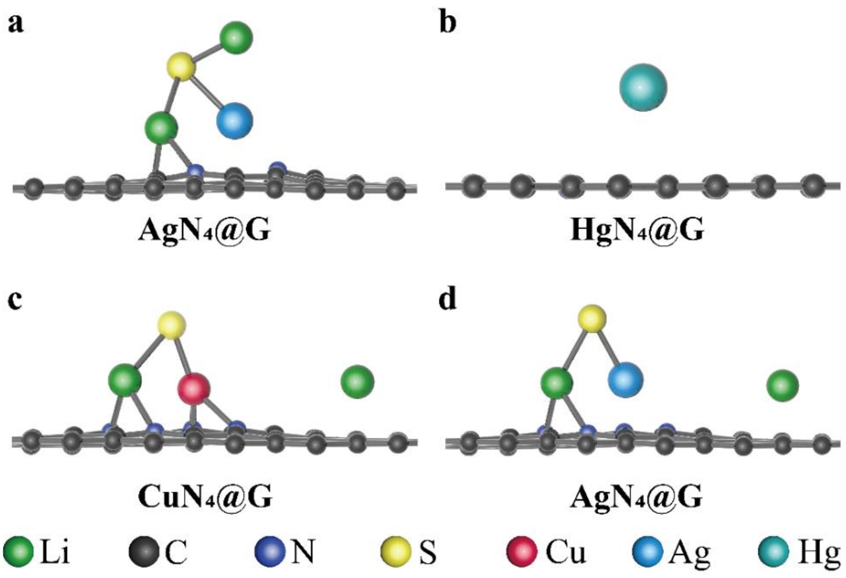

Figure S1. Lattice deformation configurations. Schematic illustrations for the atomic lattice deformation configurations before or after the deposition of adsorbate. (a) Li_2S adsorbed on AgN_4@G. (b) Fully relaxed HgN_4@G substrate. Final state of Li_2S disassociation on CuN_4@G (c) and AgN_4@G (d).

According to the optimized atomic configurations shown in Figure S1, the active center of CuN_4, AgN_4, and HgN_4 suffer from atomic deformation after or before the deposition of adsorbates, which is consist with the previous work.[7] This implies that pyridine-N center cannot firmly attach the monodisperse Cu, Ag, and Hg atoms. Hence, these three substrates are not studied further.

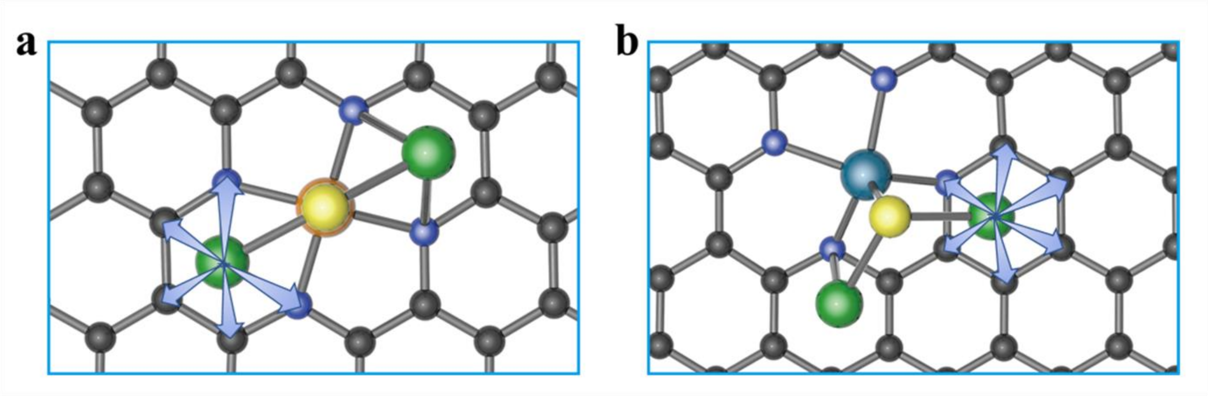

Figure S2. Stretching effect on Li atom. (a-b) Top views of two typical Li_2S adsorption patterns, where the arrows around Li atom represent the stretching effect of C and N atoms from the substrate on Li atom.

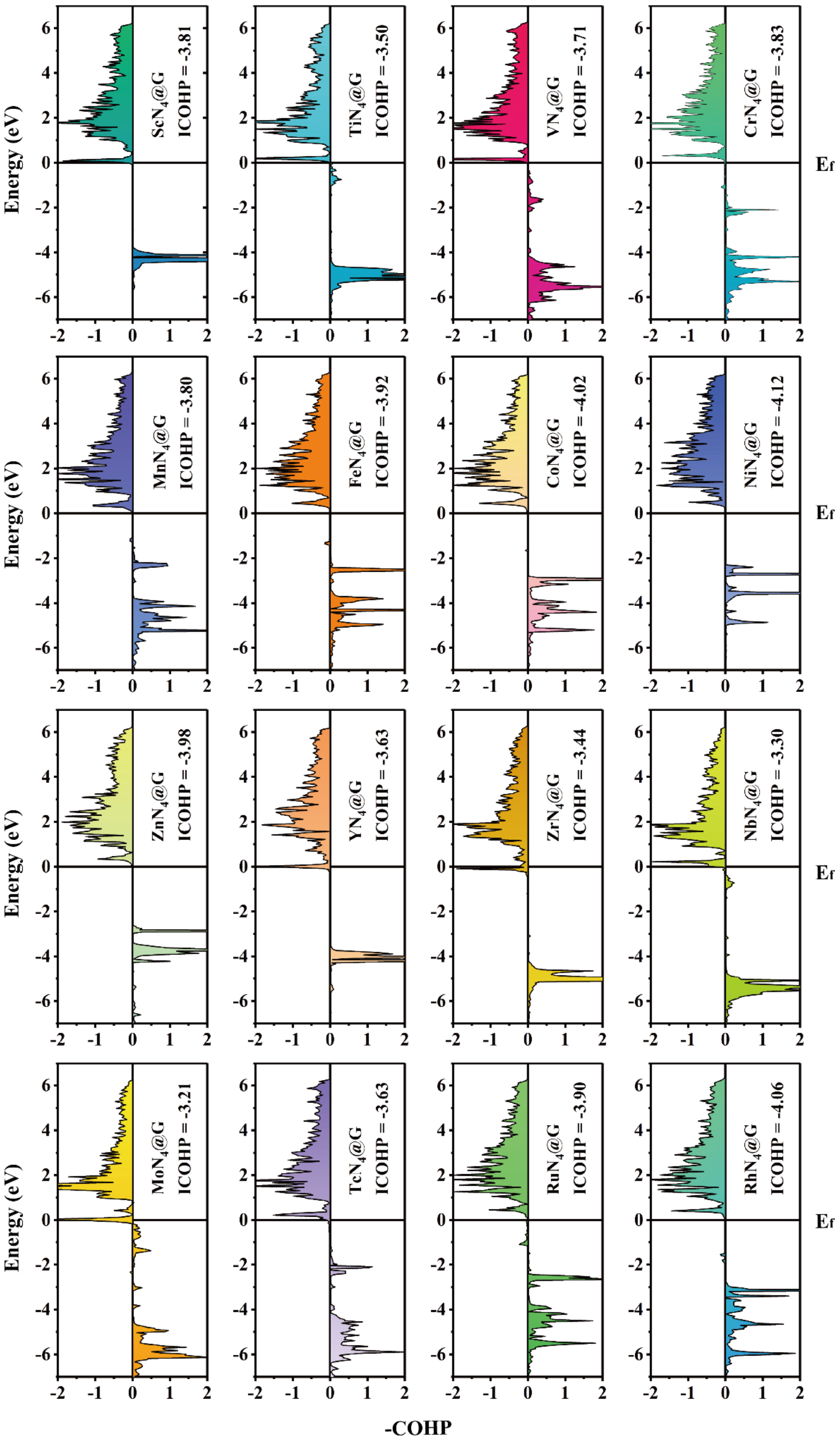
Energy (eV)
-COHP
Ef
ScN4@G ICOHP = -3.81
TiN4@G ICOHP = -3.50
VN4@G ICOHP = -3.71
CrN4@G ICOHP = -3.83
MnN4@G ICOHP = -3.80
FeN4@G ICOHP = -3.92
CoN4@G ICOHP = -4.02
NiN4@G ICOHP = -4.12
ZnN4@G ICOHP = -3.98
YN4@G ICOHP = -3.63
ZrN4@G ICOHP = -3.44
NbN4@G ICOHP = -3.30
MoN4@G ICOHP = -3.21
TcN4@G ICOHP = -3.63
RuN4@G ICOHP = -3.90
RhN4@G ICOHP = -4.06

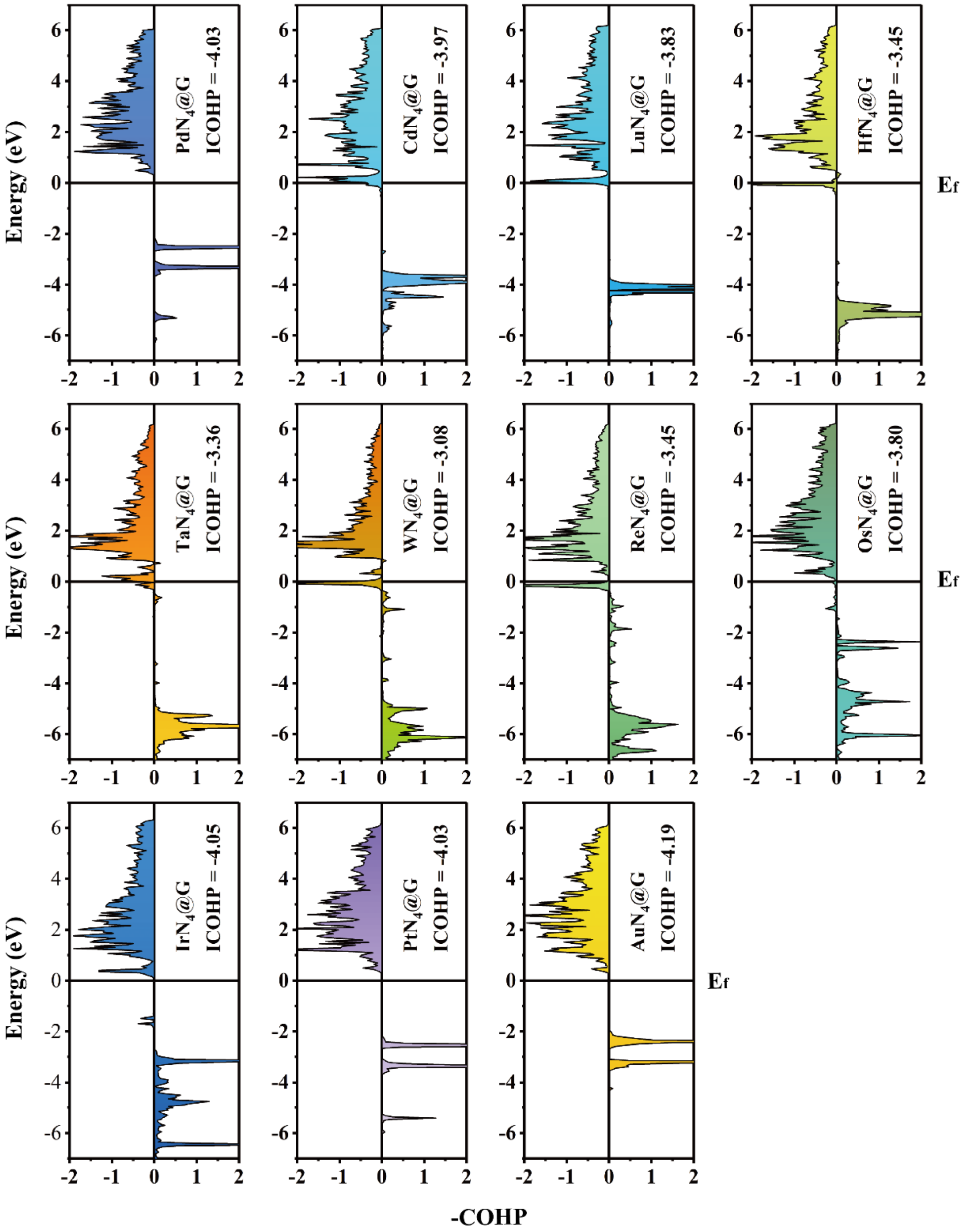

Figure S3. COHP analysis. COHP analysis of Li-S interatomic interactions of Li_2S adsorbed on MN_4@G and their corresponding ICOHP values.

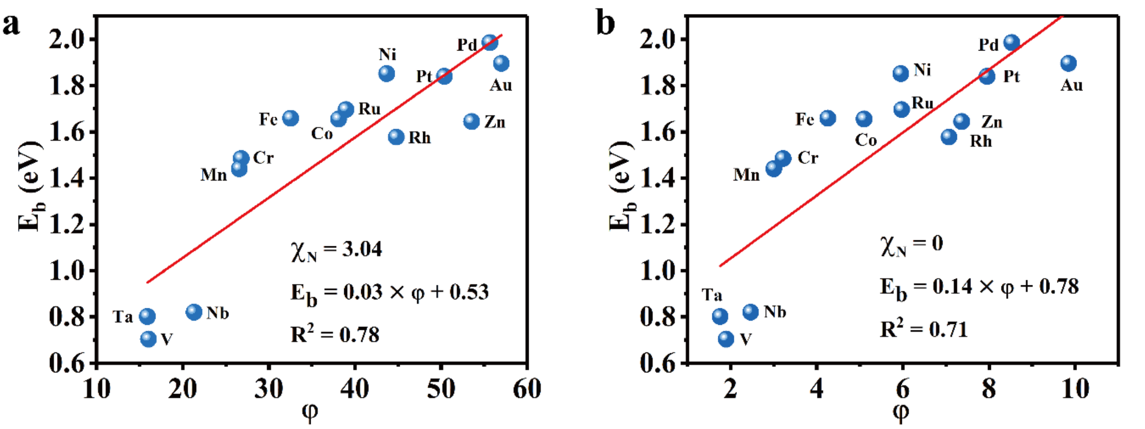

Figure S4. The modification of χ_N to the fitness of φ-E_b relationship, where χ_N = 3.04 in graph (a) represents the actual electronegativity of N, while χ_N = 0 in graph (b) means that the coordination of metal is excluded from the calculation of φ.

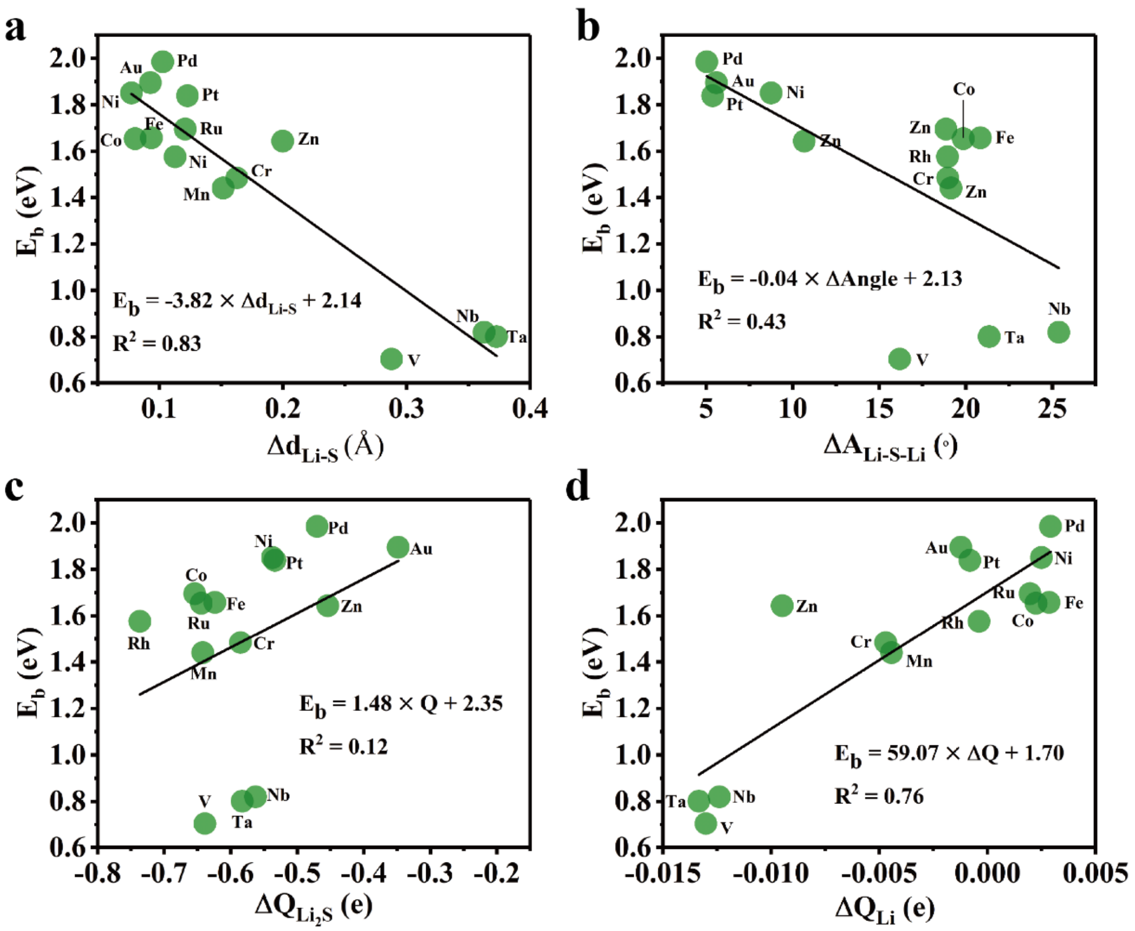

Figure S5. Potential descriptors for Li_2S decomposition. Scatterplots for the fitting relationship between E_b and the variations in (a) bond length ($\Delta d_{Li\text{-}S}$) and (b) bond angle

($\Delta A_{Li\text{-}S\text{-}Li}$), as well as in the Bader charge difference of (c) Li_2S (ΔQ_{Li2S}) and (d) Li atom (ΔQ_{Li}), before and after the deposition of Li_2S. Herein, $\Delta A_{Li\text{-}S\text{-}Li}$ is the absolute value of the corresponding data in Table S4.

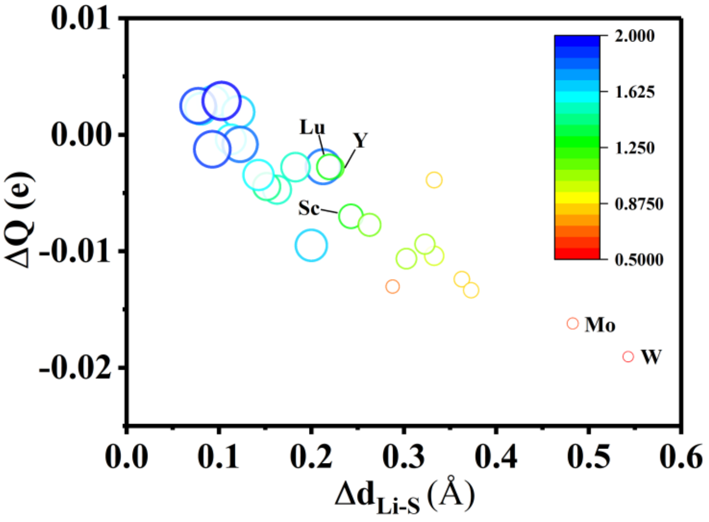

Figure S6. Adsorption activation. Adsorption activation of Li_2S after adsorbed on the SACs, where the size and color of the bubbles are classified by E_b values that are given by ΔE-E_b relationship or CI-NEB method. The horizontal and vertical coordinates represent $\Delta d_{Li\text{-}S}$ and ΔQ_{Li}, respectively.

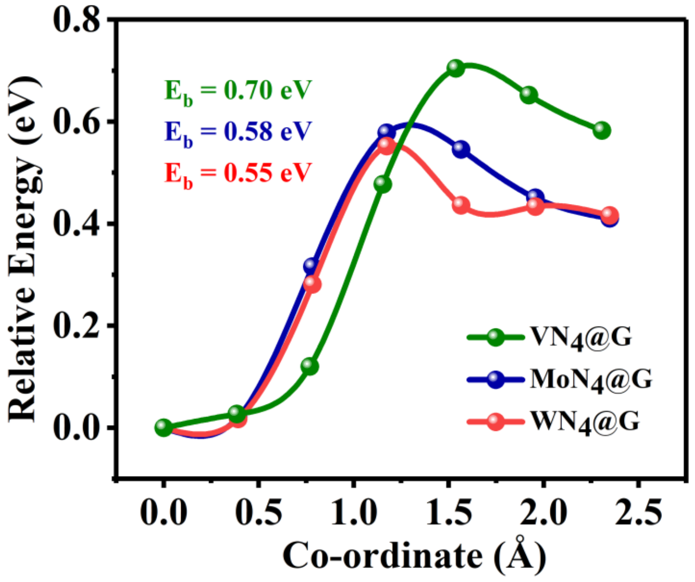

Figure S7. Li_2S decomposition process. The energy diagram of Li_2S decomposition on

$MoN_4@G$, WN4@G, and $VN_4@G$.

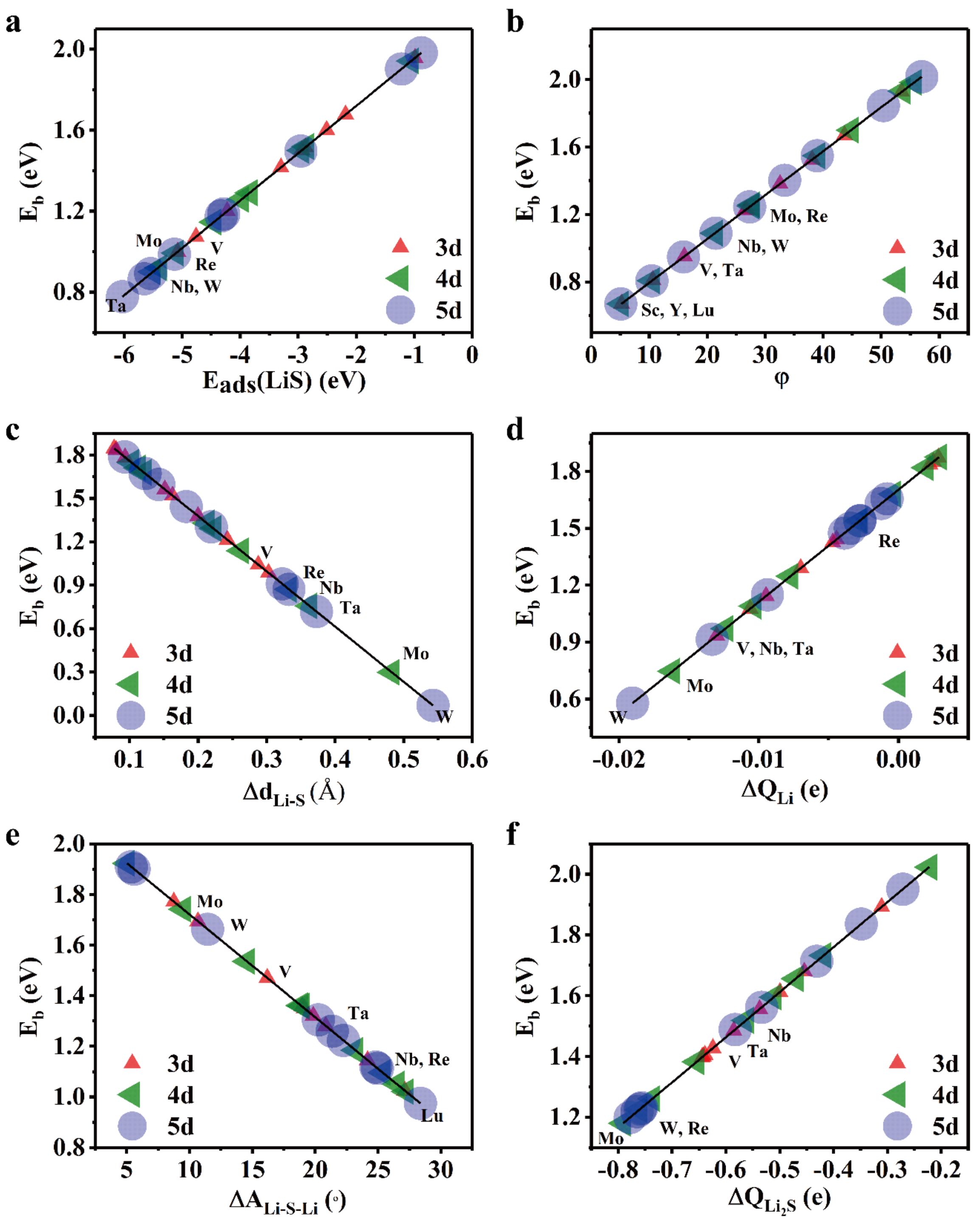

Figure S8. Prediction and screening of efficient catalysts among $MN_4@G$. Prediction and screening based on E_{ads}(LiS) (a), φ (b), $\Delta d_{Li\text{-}S}$ (c), ΔQ_{Li} (d), $\Delta A_{Li\text{-}S\text{-}Li}$ (e), and ΔQ_{Li2S} (f). The potential SACs with the best catalytic performance given by these parameters were marked in the lower left or lower right corners. The black lines are the correlation functions obtained in

previous discussion.

According to the predicted results of E_{ads}(LiS) and φ (Figure S8a and b), SACs of Ta, Sc, Y, and Lu on N_4@G are considered as the potential catalysts with the highest catalytic activity. For TaN_4@G, E_b value estimated using E_{ads}(LiS) is 0.79 eV, which is in accordance with the CI-NEB result (0.80 eV). When it comes to MoN_4@G and WN_4@G, E_b-E_{ads}(LiS) relationship gives rise to the worst prediction, 1.01 and 0.91 eV for them apiece. As for φ, the poor estimation capability is also evidenced by MoN_4@G and WN_4@G, with E_b values of 1.26 and 1.09 eV, respectively, significantly deviated from the CI-NEB results. Whereas, the decomposition barriers of Li_2S on ScN_4@G, YN_4@G, and LuN_4@G are all greater than 1.20 eV estimated using the descriptors of ΔE and ICOHP. Otherwise, the variation in bong length and Bader charge difference of Li atom is not obvious as shown in Figure S6. Therefore, we have a high degree of confidence that ScN_4@G, YN_4@G, and LuN_4@G are electrocatalytically inefficient.

Based on $\Delta d_{Li\text{-}S}$, ΔQ_{Li}, $\Delta A_{Li\text{-}S\text{-}Li}$, and ΔQ_{Li2S}, shown in Figure S8 c-f, we cannot generally screen those six SACs of WN_4@G, MoN_4@G, NbN_4@G, TaN_4@G, VN_4@G, and ReN_4@G or some of them, partially consistent with predictions by ΔE, whereas their relative trend could be reproduced by these descriptors, which could be confirmed by the data listed in Table S6.

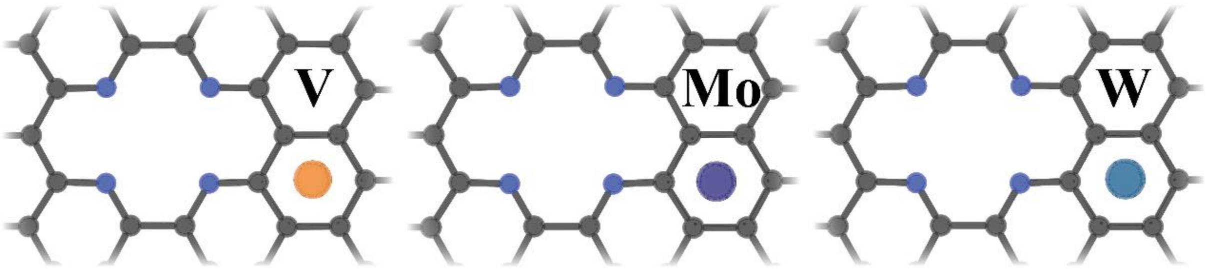

Figure S9. The calculation of stability. Atomic configurations of monodisperse TM atom adsorb at the neighboring hollow site of pyridine-N center.

Herein, the stability of the substrates was characterized by the adsorption energy difference (ΔE_{ads}) of metal atom accommodated on pyridine-N center and neighboring graphene site (Figure S9), which is calculated as $\Delta E_{ads} = E$(TM on pyridine-N center) – E(TM on neighboring site). A lower ΔE_{ads} value means a higher stability of a substrate.[8] The ΔE_{ads} of VN_4@G, MoN_4@G and WN_4@G are −6.18, −5.97, and −6.68 eV respectively, indicating that these atomic disperse metal are trapped strongly on pyridine-N center and unlikely to diffuse and aggregate.

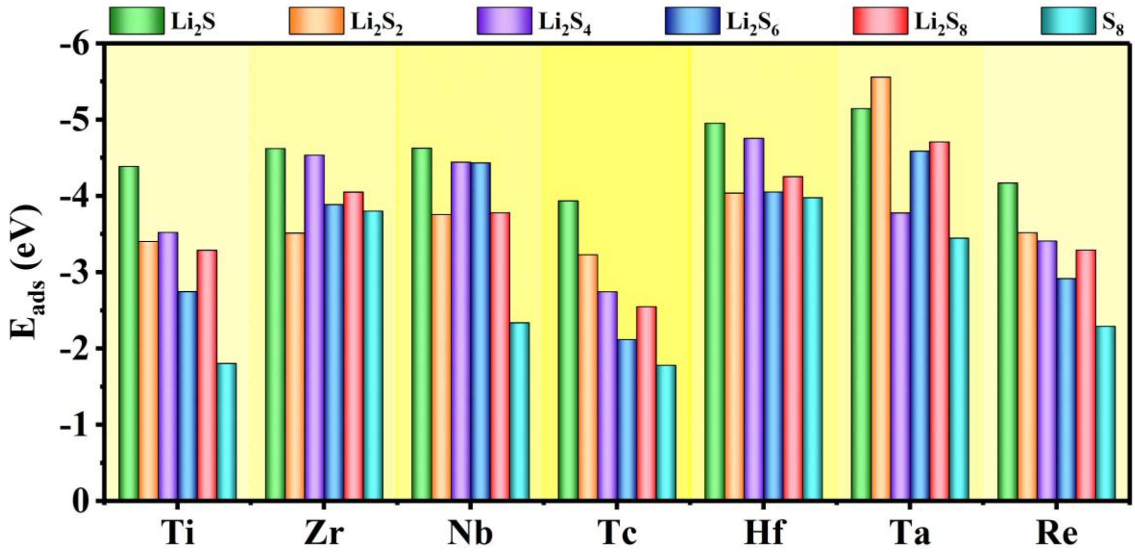

Figure S10. The adsorption energies of LiPs and S_8 on SACs of Ti, Zr, Nb, Tc, Hf, Ta, and Re, respectively.

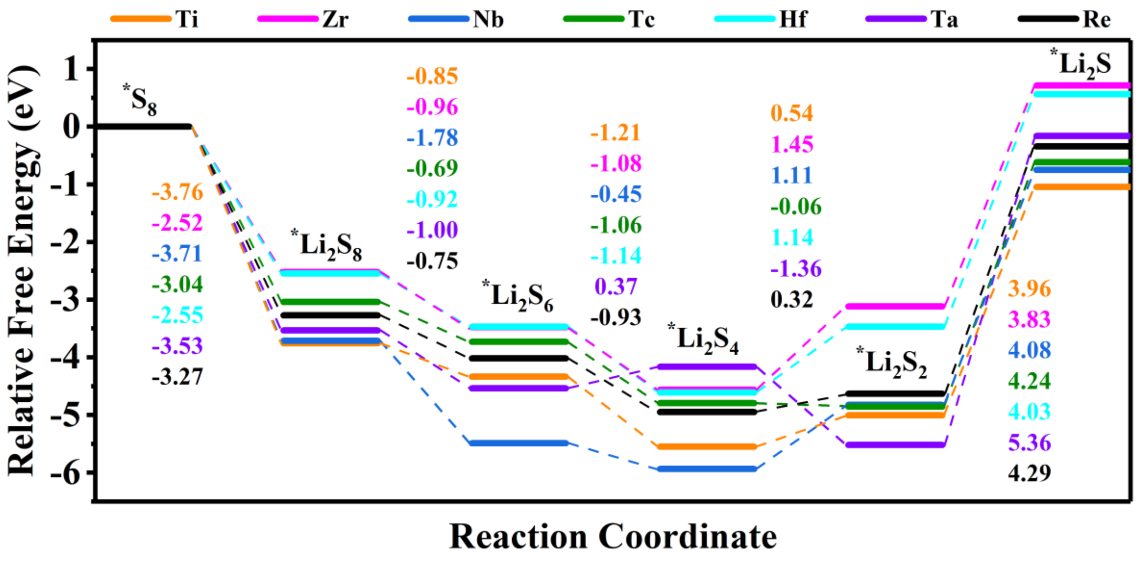

Figure S11. Relative Gibbs free energies diagram. The values represent the change of Gibbs free energy for each lithiation step.

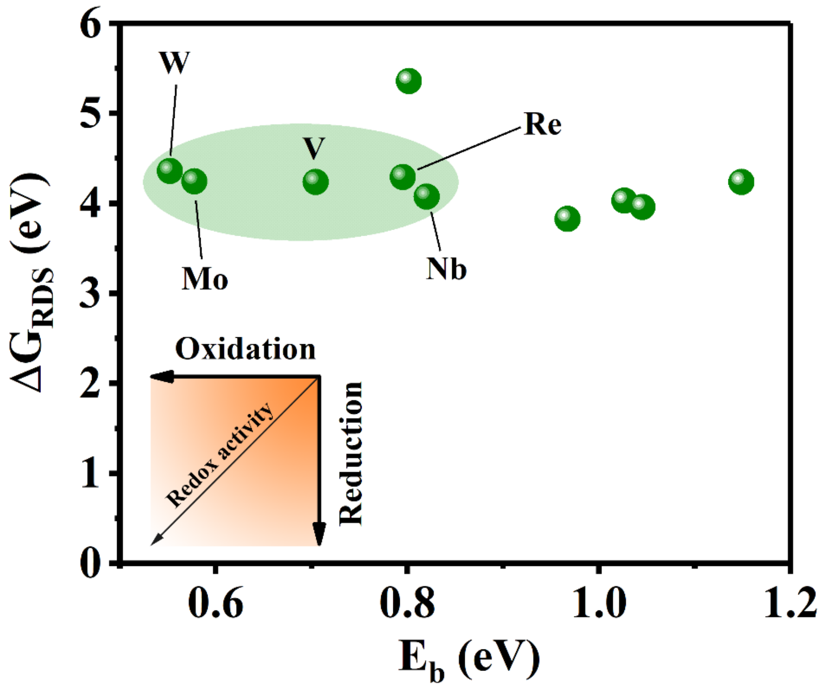

Figure S12. Redox activity. The oxidation performance was evaluated by Li_2S decomposition barrier, while the reduction performance was assessed by ΔG_{RDS}. The E_b values of the corresponding SACs of W, Mo, V, Ta, and Nb were given by CI-NEB method, while the others were generated by ΔE-E_b relationship.

Table S1. Average bond length of M-N. The average bond length between monodisperse metal and pyridine N.

single atom	$d_{M\text{-}N}$ (Å)	single atom	$d_{M\text{-}N}$ (Å)	single atom	$d_{M\text{-}N}$ (Å)
Sc	2.09	Y	2.21	Lu	2.16
Ti	2.03	Zr	2.13	Hf	2.11
V	1.95	Nb	2.09	Ta	2.07
Cr	1.95	Mo	2.04	W	2.01
Mn	1.92	Tc	1.97	Re	1.99
Fe	1.88	Ru	1.96	Os	1.94
Co	1.88	Rh	1.95	Ir	1.95
Ni	1.88	Pd	1.96	Pt	1.96
Cu	1.93	Ag	1.99	Au	1.97
Zn	1.96	Cd	2.22	Hg	3.18

Table S2. The adsorption energies of Li_2S on SACs.

single atom	E_{ads} (eV)	single atom	E_{ads} (eV)
Sc	−4.06	Ru	−3.87
Ti	−4.39	Rh	−2.81
V	−3.93	Pd	−1.33
Cr	−3.08	Ag	−2.85
Mn	−2.61	Cd	−3.39
Fe	−2.81	Lu	−4.26
Co	−2.53	Hf	−4.95
Ni	−1.47	Ta	−5.15
Cu	−1.78	W	−4.50
Zn	−2.53	Re	−4.17
Y	−3.85	Os	−4.13
Zr	−4.62	Ir	−2.89
Nb	−4.63	Pt	−1.41
Mo	−4.06	Au	−1.23
Tc	−3.94		

Table S3. ICOHP analysis. The ICOHP values of the interactions of Li-S, Li-C, Li-N, where the AVG represent the average ICOHP values of Li-C and Li-N interactions.

single atom	Li-S	Li-X (X = C, N from substrates)								
		Li-C					AVG	Li-N		AVG
V	−3.71	−0.76	−0.66	−0.72			−0.71	−1.02	−1.20	−1.11
Cr	−3.83	−0.73	−0.67	−0.67			−0.69	−0.96	−0.96	−0.96
Mn	−3.80	−0.72	−0.68	−0.69			−0.70	−0.95	−0.95	−0.95
Fe	−3.92	−0.71	−0.69	−0.70			−0.70	−0.93	−0.95	−0.94
Co	−4.02	−0.70	−0.70	−0.70			−0.70	−0.95	−0.95	−0.95
Ni	−4.12	−0.64	−0.65	−0.65			−0.65	−0.99	−0.99	−0.99
Zn	−3.98	−0.70	−0.59	−0.60			−0.63	−0.91	−0.91	−0.91
Nb	−3.30	−0.67	−0.67	−0.62	−0.59	−0.56	−0.62	−1.07		−1.07
Ru	−3.90	−0.72	−0.69	−0.69			−0.70	−0.93	−0.93	−0.93
Rh	−4.06	−0.73	−0.69	−0.69			−0.70	−0.97	−0.97	−0.97
Pd	−4.03	−0.61	−0.56	−0.55			−0.57	−1.04	−1.02	−1.03
Ta	−3.36	−0.63	−0.69	−0.67	−0.62	−0.59	−0.64	−1.02		−1.02
Pt	−4.03	−0.61	−0.61	−0.60			−0.61	−1.00	−0.99	−1.00
Au	−4.19	−0.63	−0.57	−0.58			−0.59	−0.90	−0.92	−0.91

Table S4. Morphology changes of Li_2S upon deposition. The variation in bond length and bond angle after Li_2S adsorbed on MN_4@G. Herein, the variations are calculated as the difference between adsorption state Li_2S and isolated Li_2S.

single atom	$\Delta d_{Li\text{-}S}$(Å)	$\Delta A_{Li\text{-}S\text{-}Li}$ (°)	single atom	$\Delta d_{Li\text{-}S}$(Å)	$\Delta A_{Li\text{-}S\text{-}Li}$(°)
Sc	0.24	−27.13	Ru	0.12	18.85
Ti	0.30	−24.16	Rh	0.11	18.94
V	0.29	16.18	Pd	0.10	−5.03
Cr	0.16	18.95	Cd	0.21	−26.49
Mn	0.15	19.14	Lu	0.22	−28.37
Fe	0.09	20.84	Hf	0.32	−24.83
Co	0.08	19.85	Ta	0.37	−21.35
Ni	0.08	8.75	W	0.54	11.44
Zn	0.20	10.66	Re	0.33	−24.95
Y	0.22	−27.23	Os	0.18	20.25
Zr	0.33	−23.20	Ir	0.14	22.25
Nb	0.36	−25.37	Pt	0.12	−5.37
Mo	0.48	9.49	Au	0.09	5.59
Tc	0.26	14.57			

Table S5. Prediction capability of the descriptors. E_b values given by ICOHP-, ΔE-, ΔE(*LiS)-, E_{ads}(LiS)-, φ-E_b relationships, and CI-NEB method. All data are in units of eV.

Single atom	E_b (ICOHP)	E_b (ΔE)	E_b (ΔE(*LiS))	E_b (E_{ads}(LiS))	E_b (φ)	E_b (CI-NEB)
Sc	1.46	1.48	1.46	1.20	0.67	
Ti	1.06	1.05	1.00	1.00	0.81	
V	1.33	0.83	0.88	1.07	0.95	0.70
Cr	1.48	1.45	1.41	1.42	1.23	1.48
Mn	1.45	1.40	1.32	1.50	1.23	1.44
Fe	1.61	1.65	1.54	1.52	1.38	1.66
Co	1.73	1.68	1.62	1.60	1.53	1.66
Ni	1.87	1.91	2.02	1.96	1.67	1.85
Zn	1.69	1.66	1.90	1.68	1.93	1.64
Y	1.22	1.45	1.49	1.26	0.67	
Zr	0.98	0.97	0.93	0.92	0.81	
Nb	0.80	0.79	0.83	0.90	1.09	0.82
Mo	0.68	0.61	0.69	0.99	1.26	0.58
Tc	1.22	1.15	1.15	1.15	1.25	
Ru	1.58	1.70	1.63	1.29	1.55	1.70
Rh	1.79	1.59	1.56	1.52	1.70	1.58
Pd	1.75	1.85	1.84	1.94	1.98	1.99
Cd	1.67	1.82	1.99	1.50	1.93	
Lu	1.48	1.63	1.58	1.19	0.67	
Hf	1.00	1.03	0.99	0.87	0.81	
Ta	0.87	0.77	0.83	0.78	0.95	0.80
W	0.52	0.61	0.70	0.89	1.09	0.55
Re	1.00	0.80	0.76	0.99	1.25	
Os	1.45	1.49	1.43	1.18	1.40	
Ir	1.78	1.58	1.55	1.50	1.55	
Pt	1.75	1.88	1.76	1.90	1.85	1.84
Au	1.96	1.92	1.91	1.49	2.02	1.90

Table S6. Prediction capability of the descriptors. E_b values given by $\Delta d_{Li\text{-}S}$-, ΔQ_{Li}-, $\Delta A_{Li\text{-}S\text{-}Li}$-, ΔQ_{Li2S}-E_b relationships, and CI-NEB method. All data are in units of eV.

Single atom	E_b ($\Delta d_{Li\text{-}S}$)	E_b (ΔQ_{Li})	E_b ($\Delta A_{Li\text{-}S\text{-}Li}$)	E_b (ΔQ_{Li2S})	E_b (CI-NEB)
Sc	1.22	1.29	1.03	1.89	
Ti	0.99	1.08	1.15	1.61	
V	1.04	0.93	1.47	1.41	0.70
Cr	1.52	1.43	1.36	1.48	1.48
Mn	1.56	1.44	1.35	1.40	1.44
Fe	1.79	1.87	1.28	1.43	1.66
Co	1.84	1.84	1.32	1.40	1.66
Ni	1.85	1.85	1.77	1.56	1.85
Zn	1.38	1.14	1.69	1.68	1.64
Y	1.29	1.54	1.02	2.02	
Zr	0.87	1.09	1.18	1.73	
Nb	0.76	0.97	1.10	1.52	0.82
Mo	0.30	0.75	1.74	1.18	0.58
Tc	1.14	1.25	1.54	1.22	
Ru	1.68	1.82	1.36	1.38	1.70
Rh	1.71	1.68	1.36	1.26	1.58
Pd	1.75	1.88	1.92	1.66	1.99
Cd	1.33	1.54	1.05	1.60	
Lu	1.30	1.54	0.97	1.95	
Hf	0.91	1.15	1.12	1.71	
Ta	0.72	0.92	1.26	1.49	0.80
W	0.07	0.58	1.66	1.20	0.55
Re	0.87	1.48	1.11	1.23	
Os	1.44	1.54	1.30	1.22	
Ir	1.60	1.50	1.22	1.23	
Pt	1.67	1.66	1.91	1.56	1.84
Au	1.79	1.63	1.90	1.84	1.90

Table S7. The values of E_b and ΔG_{RDS} on those SACs possessing ΔE < 1.0 eV, where the E_b values of SACs of W, Mo, V, Ta, and Nb were given by CI-NEB method, while the others were generated by ΔE-E_b relationship.

single atom	W	Mo	V	Re	Ta	Nb	Zr	Hf	Ti	Tc
E_b (eV)	0.55	0.58	0.70	0.80	0.80	0.82	0.97	1.03	1.05	1.15
ΔG_{RDS} (eV)	4.36	4.25	4.24	4.29	5.36	4.08	3.83	4.03	3.96	4.24

Supporting references

[1] G. Kresse, D. Joubert, *Phys. Rev. B* **1999**, *59*, 1758.
[2] P. E. Blöchl, *Phys. Rev. B* **1994**, *50*, 17953.
[3] J. P. Perdew, K. Burke, M. Ernzerhof, *Phys. Rev. Lett.* **1996**, *77*, 3865.
[4] K. Lee, É. D. Murray, L. Kong, B. I. Lundqvist, D. C. Langreth, *Phys. Rev. B* **2010**, *82*, 081101.
[5] D. J. Chadi, *Phys. Rev. B* **1977**, *16*, 1746.
[6] Q. He, B. Yu, H. Wang, M. Rana, X. Liao, Y. Zhao, *Nano Res.* **2020**, *13*, 2299.
[7] G. Zhou, S. Zhao, T. Wang, S.-Z. Yang, B. Johannessen, H. Chen, C. Liu, Y. Ye, Y. Wu, Y. Peng, C. Liu, S. P. Jiang, Q. Zhang, Y. Cui, *Nano Lett.* **2020**, *20*, 1252.
[8] W. Zhao, L. Zhang, Q. Luo, Z. Hu, W. Zhang, S. Smith, J. Yang, *ACS Catal.* **2019**, *9*, 3419.
